# Efficacy and safety of follitropin delta for ovarian stimulation in vitro fertilization/ intracytoplasmic sperm injection cycles: a systematic review with meta-analysis

**DOI:** 10.1186/s13048-024-01372-w

**Published:** 2024-03-14

**Authors:** Stefano Palomba, Donatella Caserta, Paolo Emanuele Levi-Setti, Andrea Busnelli

**Affiliations:** 1 Unit of Gynecology, Department of Surgical and Medical Sciences and Translational Medicine, Sant’Andrea Hospital, Sapienza University of Rome, Rome, Italy; 2 Department of Biomedical Sciences, Humanitas University, Pieve Emanuele, Milan, Italy; 3 Department of Gynecology and Reproductive Medicine, IRCCS Humanitas Research Hospital, Rozzano, Milan, Italy

**Keywords:** Follitropin delta, Gonadotropin, Infertility, IVF, Ovarian stimulation, Sterility

## Abstract

**Background:**

Follitropin delta is a novel recombinant follicle stimulating hormone preparation uniquely expressed in a human fetal retinal cell line by recombinant DNA technology. To date, no systematic review was available about the safety and the efficacy of the follitropin delta. The objective of this study was systematically reviewing the available literature and to provide updated evidence regarding the efficacy-safety profile of follitropin delta when compared to other gonadotropin formulations for ovarian stimulation in in vitro fertilization (IVF) and intracytoplasmic sperm injection (ICSI) cycles.

**Methods:**

An extensive search was performed to identify phase 1, phase 2 and phase 3 RCTs in humans focused on follitropin delta use for ovarian stimulation in IVF/ICSI cycles. The risk of bias and the overall quality of the evidence was analyzed. All data were extracted and analyzed using the intention-to-treat principle and expressed per woman randomized.

**Results:**

A total of 7 RCTs (1 phase 1 RCT, 2 phase 2 RCTs and 4 phase 3 RCTs) were included in the qualitative analysis, whereas data of three phase 3 RCTs were meta-analyzed. All trials compared personalized recombinant follitropin delta treatment versus conventional recombinant follitropin alfa/beta administration in potentially normo-responder patients who receive ovarian stimulation in GnRH antagonist IVF/ICSI cycles. No difference between two regimens was detected for clinical pregnancy rate [odds ratio (OR) 1.06; 95% confidence intervals (CI): 0.90, 1.24; *P* = 0.49; I^2^ = 26%], ongoing pregnancy rate (OR 1.15; 95%CI: 0.90, 1.46; *P* = 0.27; I^2^ = 40%), and live birth rate (OR 1.18; 95%CI: 0.89, 1.55; *P* = 0.25; I^2^ = 55%). No data were available regarding cumulative success rates. The rate of adoption of strategies to prevent ovarian hyperstimulation syndrome (OHSS) development (OR 0.45; 95%CI: 0.30, 0.66; *P* < 0.0001; I^2^ = 0%), and the rate of both early OHSS (OR 0.62; 95%CI: 0.43, 0.88; *P* = 0.008; I^2^ = 0%) and all forms of OHSS (OR 0.61; 95%CI: 0.44, 0.84; *P* = 0.003; I^2^ = 0%) were significantly lower in the group of patients treated with personalized follitropin delta treatment compared to those treated with conventional follitropin alfa/beta administration.

**Conclusion:**

Personalized follitropin delta treatment is associated with a lower risk of OHSS compared to conventional follitropin alfa/beta administration in potentially normo-responder patients who receive ovarian stimulation in GnRH antagonist IVF/ICSI cycles. The absence of cumulative data does not allow definitive conclusions to be drawn regarding the comparison of the effectiveness of the two treatments.

**Protocol study registration:**

CRD42023470352 (available at http://www.crd.york.ac.uk/PROSPERO).

## Introduction

In vitro fertilization (IVF) and intracytoplasmic sperm injection (ICSI) are infertility treatments widely used, effective and with a low rate of complications [1]. One of the key factors affecting the success rate of the procedures is the ovarian stimulation with exogenous gonadotropins [2–4]. Available evidence demonstrates a positive linear correlation between the number of oocytes retrieved and the IVF success rates. A rapid increase in live birth rate (LBR) with the number of oocytes retrieved to approximately 16–20 oocytes, at which point it continued to increase but with diminishing returns, has been demonstrated [5]. The goal of maximizing the number of good quality oocytes retrieved must be balanced against the risk of ovarian hyperstimulation syndrome (OHSS). Several strategies have been developed over the years to reduce the risk of developing this complication [6]. The most effective was the introduction of gonadotropin releasing hormone (GnRH) antagonists to suppress the luteinizing hormone (LH) surge, and of GnRH agonist triggering followed by a “freeze all” policy [7]. This strategy is widely accepted for presumed high-responder patients [6, 8]. However, many OHSS cases occur in presumed normal responders, and the clinical and scientific interest for interventions aimed to minimize OHSS is thus still high.

A recent umbrella review, aimed to identify the best evidence-based interventions to prevent or reduce the incidence and severity of OHSS in patients undergoing IVF/ICSI cycles, intercepted a total of 28 systematic reviews of randomized controlled trials (RCTs) with meta-analysis [9]. Its results confirmed the efficacy of GnRH antagonist IVF/ICSI cycles with GnRH agonist triggering with embryo freezing for high-risk OHSS patients. Authors also showed that the progestin-primed ovarian stimulation protocol is a valid option in case of elective embryo transfer (ET) as well as for cancer patients in the context of fertility preservation and for donors [9]. The use of mild gonadotropin stimulation, the metformin coadministration and dopamine agonists treatment resulted also effective strategies for high-risk patients who receive GnRH agonist down-regulation [9]. Novel interventions, not still formally included in systematic reviews due to the paucity of data, also deserve to be mentioned and should not be underestimated [10]. This is the case of follitropin delta that resulted, notwithstanding the lack of type A evidence, an effective treatment in terms of reduction of early OHSS risk in GnRH antagonist IVF/ICSI cycles with potentially better reproductive outcomes when compared with follitropin alfa [11].

Follitropin delta is a novel recombinant follicle stimulating hormone (r-FSH) preparation uniquely expressed in a human fetal retinal cell line (PER.C6VR) by recombinant DNA technology [12]. It shows a specific glycosylation profile characterized by an α2.3 and α2.6-linked sialic acid sugar chain [13]. The amino acid sequences of the two FSH subunits α and β are identical to the endogenous human FSH sequences with α2,6-linked sialic acid and bisecting N-acetylglucosamine [14]. This glycosylation profile induces a lower clearance and a higher ovarian response in humans than other r-FSH preparations when administered at equal doses of biological activity [12, 14]. An individualized dosing algorithm for follitropin delta incorporating body weight and pretreatment anti-Mullerian hormone (AMH) levels has been developed [15].

Although promising, follitropin delta for ovarian stimulation is supported by isolated RCTs, conducted in different settings and which are focused on different phases of pharmacological research. Against that background, we felt it was time to provide clinicians with a data synthesis. Specifically, the aim of this systematic review and meta-analysis was to provide updated evidence regarding both the efficacy (both in terms of ovarian response and reproductive outcomes) and the safety of follitropin delta when compared to other gonadotropin formulations for ovarian stimulation in IVF/ICSI cycles.

## Methods

The protocol of the current review was registered on the PROSPERO website (Protocol study registration: PROSPERO CRD42023470352, available at http://www.crd.york.ac.uk/PROSPERO), and followed the Preferred Reporting Items for Systematic Reviews and Meta-Analyses (PRISMA) 2020 statement [16] (http://www.prisma-statement.org) and the Population, Intervention, Comparison, Outcome (PICO) model [17]. No formal ethical approval was required because the study did not involve humans and/or the use of human tissue and/or hospital records samples, and no personal data were recorded and analyzed.

### Review question

The primary question of the current systematic review with meta-analysis was: is the safety-efficacy profile of follitropin delta better than those of the other gonadotropins for ovarian stimulation in IVF/ICSI cycles?

#### PICO model

According to the PICO model [17], “Population” included women who undergo IVF/ICSI treatments, “Intervention” was considered the use of follitropin delta to stimulate multiple ovulation, “Comparison” included another gonadotropin treatment or another potentially active intervention, and “Outcome” included primary and secondary outcomes of safety and efficacy. Primary outcomes (critical) for the safety issues were considered the incidence of maternal death and of hospital admission, whereas for efficacy issues were the cumulative LBR (CLBR), the LBR and the number of oocytes retrieved. Secondary outcomes included: incidence and severity of OHSS (important), days of hospitalization for complications (important), pregnancy complications (important), neonatal and offspring outcomes (important) as safety endpoints, and cumulative clinical pregnancy rate (CCPR, important), CPR (important), pregnancy rate (important), cumulative ongoing pregnancy rate (COPR, important), OPR (important), implantation rate (important), miscarriage rate (important), and number of mature oocytes (important) as efficacy endpoints. All other outcomes (including total dose of gonadotropins, duration of ovarian stimulation, number of embryos obtained, biochemical pregnancy rate, and so on) were considered of limited importance. Outcomes were defined according to The International Glossary on Infertility and Fertility Care [18]. To assess and classify the importance of any outcome we referred to https://gdt.gradepro.org/app/handbook/handbook.html#h.1i2bwkm8zpjo.

### Data sources, search strategy, and eligible criteria

The search was performed using the key words “FE 999049” or “follitropin delta” or “gonadotropin delta” or “rekovelle” AND “in vitro fertilization” or “IVF” or “intracytoplasmic sperm injection” or “ICSI” or “assisted reproductive technology” or “ART” in the following electronic databases: Pub-Med, The Cochrane Library, EMBASE, Scopus and Web of Science. Protocols for clinical trials were searched on ClinicalTrials.gov, on clinicaltrialsregister.eu and on the national/international registries included on the World Health Organization (WHO) International Trials registry platform (available at https://www.who.int/clinical-trials-registry-platform/network/primary-registries). The authors also hand-searched the reference lists of the included articles and of previous reviews to find additional data of interest for the aim of the present study, whereas unpublished studies were not specifically sought. All publications were searched without time limits and the searches re-run prior to final analysis (October 20th, 2023).

Phase 1, phase 2 and phase 3 RCTs in humans focused on follitropin delta use for ovarian stimulation in IVF/ICSI cycles were eligible. The search was restricted to female gender and English language. Cross-over RCTs, prospective quasi-randomized and non-randomized controlled studies, controlled/uncontrolled observational and retrospective cohort studies, review articles, case reports, conference abstracts, and study protocols were excluded from final analysis. No attempt was made for searching and identifying gray literature.

### Data collection process

Two authors (SP, AB) performed, extracted, and tabulated all searches independently in duplicate. The studies retrieved by the literature search were sequentially screened for inclusion according to titles and abstracts and then to full text. For each specific intervention, a custom table to extract data was created to extract data. Data extracted and tabulated included the first author, year of publication, journal, country, study design, population characteristics, sample size, ovarian stimulation protocols, primary and secondary outcomes, and the certainty of evidence (CoE). The other authors (DC, PELS) checked the trial reports and eligibility. Any disagreements were resolved by discussion among all reviewers.

Where there was insufficient information in the included study, no attempt was made to obtain original or further data by contacting corresponding authors, it was assumed that missing participants had failed to achieve specific outcome and had not suffered reported adverse events.

### Risk of bias and quality of evidence assessment

Two authors (S.P. and A.B.) independently assessed the included studies for risks of bias using the Cochrane ’Risk of bias’ assessment tool for RCTs [19].

The overall quality of the evidence was graded according to the Grading of Recommendations Assessment, Development and Evaluation (GRADE) Working Group guidelines using GRADE’s official GRADEpro software tool (www.gradepro.org/). The GRADE approach classifies the certainty of evidence into one of four grades: high, moderate, low, and very low. For each study, a qualitative analysis was performed using the data reported in the original manuscript.

### Data synthesis

All data extracted from RCTs were analyzed using the intention-to-treat (ITT) principle and all outcomes were expressed per woman randomized. Where only data ‘per cycle’ were available, and participants had contributed multiple cycles, data were omitted from meta-analysis. Pairwise meta-analyses were performed using the fixed-effects model with the Mantel–Haenszel method. In accordance with the Cochrane guidance on Systematic Reviews [19], data relevant for the experimental intervention (follitropin delta) were combined into a single group and compared with the combined data for the comparator intervention (another gonadotropin treatment or another potentially active intervention) group during the analysis. The effect size for dichotomous outcomes was measured by calculating the odds ratio (OR). Uncertainty was expressed using 95% confidence intervals (CI). For continuous data, means and standard deviations were abstracted, and the mean difference was calculated.

Review Manager (Review Manager, RevMan, version 5.4; The Cochrane Collaboration, 2020. Available at revman.cochrane.org) was used to analyze the extracted data from the included studies. According to the Cochrane Handbook for Systematic Reviews of Intervention, an I^2^ value of 0 indicates no observed heterogeneity, whereas I^2^ values from 30 to 60% may represent moderate heterogeneity, I^2^ values from 50 to 90% may represent substantial heterogeneity, and I^2^ values from 75 to 100% represent considerable heterogeneity. The risk estimates were combined in a meta-analysis using a fixed effects model when the heterogeneity found among the studies was absent to moderate (0%≤I^2^ < 30%). When heterogeneity was moderate, substantial, or considerable (I^2^ ≥ 30%), we used the DerSimonian and Laird method for a random-effects model [20]. Statistical significance was set at *P* ≤ 0.05.

## Results

**Fig. 1:**
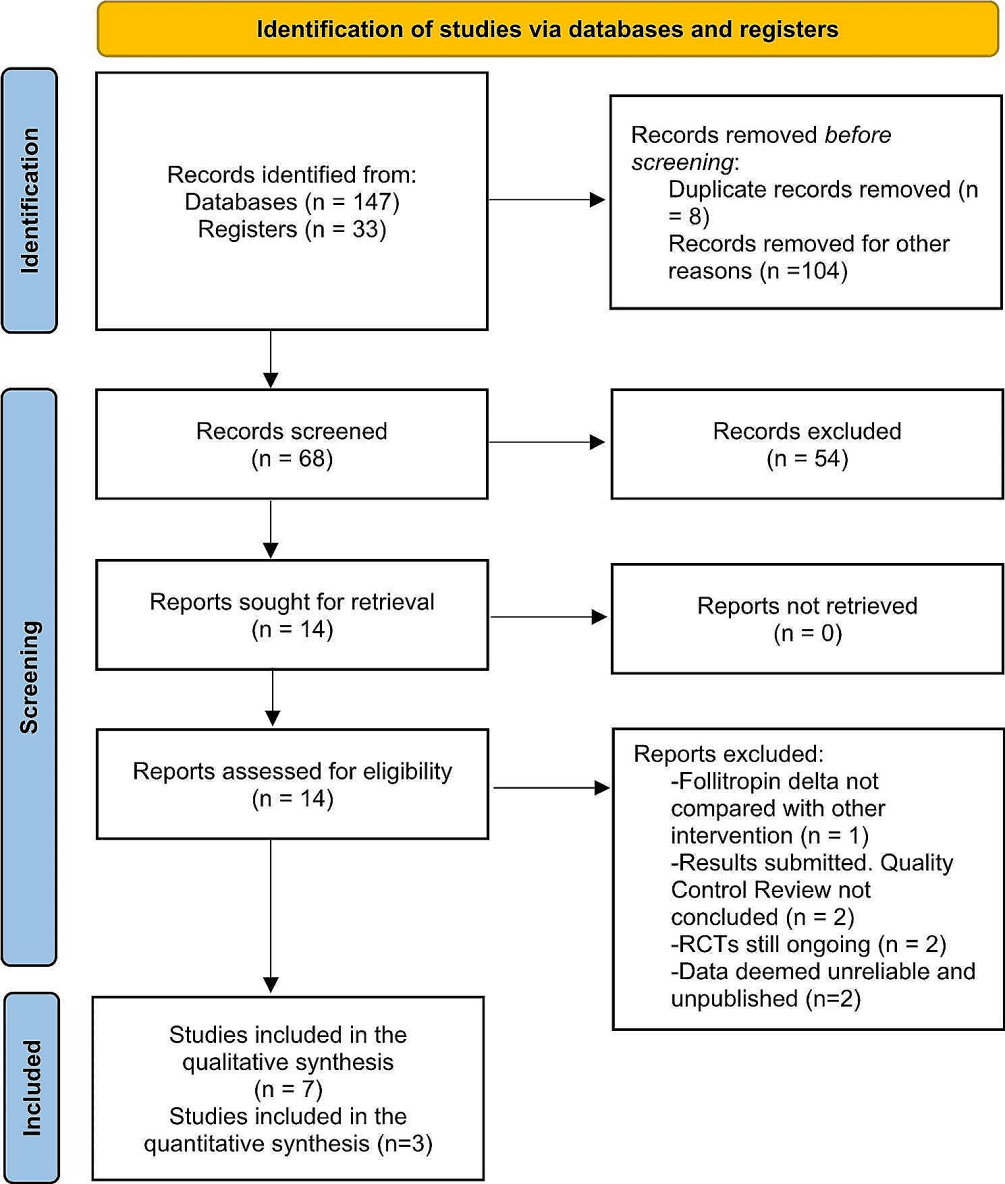
PRISMA 2020 flow diagram systematic reviews

The Fig. 1 summarizes the process of literature identification and selection of studies [16]. Our literature searches yielded 180 studies, of which 8 duplicates were removed. After a full review of titles and abstracts, 13 studies were identified as potentially eligible for inclusion, and were reviewed. One RCT was excluded because follitropin delta was not compared with other intervention (focused on the effect of choriogonadotropin beta) [21]. Two RCTs were excluded because, although their results were submitted to clinicaltrials.gov, have not yet completed the quality control review process (NCT03740737, RITA-1; and NCT03738618, RITA-2). Two RCTs were excluded because are still ongoing (NCT05103228; and NCT05263388, ADAPT-1). Two RCTs were not included because their data were deemed unreliable and were not published (NCT03809429; and 2017-003810-13).

The remaining 7 RCTs (1 phase 1 RCT, 2 phase 2 RCTs and 4 phase 3 RCTs) were included in the qualitative analysis [15, 22–27]. Data of three phase 3 RCTs were meta-analyzed [15, 25, 26]. Characteristics of included studies are reported in Table 1. All RCTs reported comparative data between different doses of follitropin delta [22, 25, 27] or between follitropin delta and follitropin alfa/beta [15, 23, 24, 26].

**Table 1:** RCTs selected and included in the systematic review (qualitative analysis)

Study	Sponsor	Study design	Setting	Study period	Nr. of included subjects	Age of study participants	Main inclusion and exclusion criteria	Interventions	Primary endpoint	Synthesis of results (primary endpoint)
Arce et al., 2014 [22]	Ferring Pharmaceuticals	Randomized, controlled, assessor-blinded, AMH-stratified (low: 5.0–14.9 pmol/L; high: 15.0–44.9 pmol/L) trial (Phase 2 trial)	Seven IVF centers in four countries (Belgium, Czech Republic, Denmark, and Spain)	From September 2011 to May 2013	A total of 265 eligible patients were randomized, with a distribution of 56% (*n* = 148) and 44% (*n* = 117) in the high and low AMH stratum, respectively	18–37 years	Inclusion criteria: women scheduled for IVF/ICSI for tubal infertility, unexplained infertility, infertility related to endometriosis stage I/II, or for male factor infertility; BMI between 18.5 and 32.0 kg/m^2^; infertility for at least 1 year; regular menstrual cycles; uterus consistent with expected normal function; presence and adequate visualization of both ovaries, without evidence of significant abnormality; early follicular phase FSH serum concentration of 1–12 IU/L and total antral follicle count ≥ 6 and ≤ 25 for both ovaries combined; serum AMH concentration of 5.0–44.9 pmol/L	On day 2–3 of the menstrual cycle, patients were randomly assigned, in a 1:1:1:1:1:1 ratio, to receive fixed daily SC injections of either 5.2 mg, 6.9 mg, 8.6 mg, 10.3 mg, or 12.1 mg of follitropin delta (FE 999,049; Ferring Pharmaceuticals), or 11 mg (150 IU) of follitropin alfa (Gonal-F® filled by mass; Merck Serono)	Number of oocytes retrieved	The number of oocytes retrieved increased in a dose–dependent manner, from 5.2 ± 3.3 oocytes with 5.2 mg/d to 12.2 ± 5.9 with 12.1 mg/d. The slopes of the dose–response curves differed significantly between the two AMH strata
Nyboe Andersen et al., 2017 [15]	Ferring Pharmaceuticals	Randomized, assessor-blinded, noninferiority trial (Phase 3 trial)	Thirty-seven IVF centers in 11 countries (Belgium, Brazil, Canada, Czech Republic, Denmark, France, Italy, Poland, Russia, Spain, and United Kingdom)	From October 8, 2013 to May 11, 2015, with live birth follow-up completed on January 11, 2016	A total of 1329 eligible women were randomized. 1326 were exposed to study drug: 665 to individualized follitropin delta and 661 to conventional follitropin alfa	18–40 years	Inclusion criteria: women scheduled for IVF/ICSI for tubal infertility, unexplained infertility, infertility related to endometriosis stage I/II, or for male factor infertility; BMI between 17.5 and 32.0 kg/m^2^; regular menstrual cycles of 24–35 days; presence of both ovaries; early follicular phase FSH serum concentration 1–15 IU/L. Exclusion criteria: endometriosis stage III–IV; history of recurrent miscarriage; use of hormonal preparations (except for thyroid medication) during the last menstrual cycle before randomization	Follitropin delta (AMH < 15 pmol/L: 12 mg/d; AMH ≥ 15 pmol/L: 0.10–0.19 mg/kg/d; maximum 12 mg/d), or follitropin alfa (150 IU/d for 5 days with potential subsequent dose adjustments up to 450 IU/d)	Ongoing pregnancy and ongoing implantation rates	Individualized follitropin delta was noninferior to conventional follitropin alfa for the primary efficacy endpoints
Bosch et al., 2019 [23]	Ferring Pharmaceuticals	Randomized, controlled, assessor-blinded trial (Phase 3 trial)	Thirty-two IVF centers in 10 countries: Belgium, Brazil, Canada, Czech Republic, Denmark, Italy, Poland, Russia, Spain, and the UK	From The trial was conducted between 26 March 2014 and 26 June 2015, with live birth follow-up completed on 26 January 2016.	In cycle 2, 513 women were enrolled and exposed; 252 to follitropin delta and 261 to follitropin alfa. In cycle 3, 189 women were enrolled, of whom 188 were exposed; 95 to follitropin delta and 93 to follitropin alfa.	18–40 years	Inclusion criteria: infertile patients who had participated in cycle 1 (Nyboe Anderson et al., 2017) and failed to achieve an ongoing pregnancy were eligible for cycle 2 and women who failed to achieve an ongoing pregnancy in cycle 2 were eligible for cycle 3. Exclusion criteria: patients with severe OHSS in a previous cycle, or patients with any clinically relevant change to any of the eligibility criteria or any clinically relevant medical history since the previous cycle.	The participating patients had in cycle 1 been randomized 1:1 to treatment with either follitropin delta (FE 999,049, Ferring Pharmaceuticals) or follitropin alfa (Gonal-F®, Merck Serono) and remained on the same gonadotrophin in cycles 2 and 3.	Proportion of women with treatment-induced anti-FSH antibodies after one and two repeated cycles of ovarian stimulation with follitropin delta	The incidence of treatment-induced anti-FSH antibodies with follitropin delta was 0.8% and 1.1% in cycles 2 and 3, respectively, which was similar to the incidence in cycle 1 (1.1%). No antibodies were of neutralizing capacity
Qiao et al., 2021 [24]	Ferring Pharmaceuticals	Randomized, controlled, assessor-blind, parallel groups, multi-center, non-inferiority trial (Phase 3 trial)	Twenty-six IVF centers in four countries/regions: mainland China, South Korea, Taiwan and Vietnam	From 1 December 2017 to 3 January 2020, with pregnancy follow-up completed on 1 September 2020	A total of 1009 women were randomized and exposed, of whom 499 were treated with follitropin delta in its individualized fixed-dose regimen and 510 with follitropin alfa in a conventional and adjustable dosing regimen.	20–40 years	Inclusion criteria: Asian reproductive-aged women scheduled for their first ovarian stimulation cycle for IVF/ ICSI for tubal infertility, unexplained infertility, endometriosis stage I/II or for male factor infertility; regular menstrual cycles of 24–35 days; presence of both ovaries; follicular phase FSH serum levels of 1–15 IU/L; BMI between 17.5 and 32.0 kg/m^2^. Exclusion criteria: women with endometriosis stage III/IV; history of recurrent miscarriage; women with one or more follicles ≥ 10 mm observed prior to randomization.	The follitropin delta treatment consisted of a fixed daily dose individualized according to each patient’s initial AMH level and body weight (AMH < 15 pmol/L: 12 µg; AMH ≥ 15 pmol/L: 0.19 to 0.10 µg/kg; min-max 6–12 µg). The follitropin alfa dose was 150 IU/day for the first 5 days with subsequent potential dose adjustments according to individual response.	Ongoing pregnancy rate	Individualized follitropin delta was noninferior to conventional follitropin alfa for the ongoing pregnancy rate (31.3% vs. 25.7%, respectively)
Ishihara et al., 2021 [25]	Ferring Pharmaceuticals	Randomized, controlled, assessor-blinded, AMH-stratified (low 5.0–14.9 pmol/L; high 15.0–44.9 pmol/L) dose-response trial (Phase 2 trial)	Ten IVF centers in Japan	From December 15, 2014 to December 29, 2015, with pregnancy follow-up data completed on October 12, 2016.	A total of 159 women were randomized, of whom 158 were exposed: 117 in the follitropin delta groups (37 in 6 µg/d, 40 in 9 µg/d, and 40 in 12 µg/d) and 41 in the 150 IU/d follitropin beta group	20–39 years	Inclusion criteria: Japanese women eligible for IVF/ICSI with tubal infertility, unexplained infertility, or infertility related to endometriosis stage I/II or with partners diagnosed with male factor infertility; BMI between 17.5 and 32.0 kg/m^2^; regular menstrual cycles of 24–35 days; presence of both ovaries; AMH: 5.0–44.9 pmol/L; early follicular phase FSH of 1–12 IU/L. Exclusion criteria: endometriosis stage III/IV; 3 or more controlled ovarian stimulation cycles for IVF/ICSI; history of recurrent miscarriage; use of hormonal preparations (except for thyroid medication) during the last menstrual cycle before randomization	Ovarian stimulation with 6, 9, or 12 mg/d of follitropin delta or 150 IU/d follitropin beta as a reference arm in a gonadotropin-releasing hormone antagonist cycle	Number of oocytes retrieved	A significant dose-relation was established between follitropin delta doses and oocytes retrieved (mean number ± SD; 7.0 ± 4.1, 9.1 ± 5.6, and 11.6 ± 5.6 for 6 µg/d, 9 µg/d, and 12 µg/d follitropin delta groups respectively) That finding remained significant within each AMH strata
Ishihara and Arce, 2021 [26]	Ferring Pharmaceuticals	Randomized, controlled, assessor-blind, multicenter, non-inferiority trial (Phase 3 trial)	17 investigational sites in Japan	Trial conducted between 7 July 2017 and 11 September 2018	A total of 347 Japanese women were randomized and exposed to ovarian stimulation, of which 170 were treated with individualized follitropin delta treatment and 177 with conventional follitropin beta treatment	20–40 years	Inclusion criteria: Japanese women scheduled to first IVF/ICSI cycle for tubal infertility, unexplained infertility or infertility related to endometriosis stage I/II, or for a partner diagnosed with male factor infertility; BMI between 17.5 and 32.0 kg/m2; regular menstrual cycles of 24–35 days; presence of both ovaries; early follicular phase FSH: 1–15 IU/l. Exclusion criteria: endometriosis stage III/IV; history of recurrent miscarriage; use of hormonal preparations (except for thyroid medication) during the last menstrual cycle before randomization	Women were randomized to individualized follitropin delta (AMH < 15 pmol/L; AMH ≥ 15 pmol/L) or conventional follitropin beta (150 IU/day for the first 5 days, with potential subsequent dose adjustments)	Number of oocytes retrieved with a pre-specified non-inferiority margin (-3.0 oocytes)	The number of oocytes retrieved after individualized follitropin delta treatment and conventional follitropin beta treatment are similar (9.3 versus 10.5; lower boundary of 95% CI: −2.3)
Shao et al., 2023 [27]	Ferring Pharmaceuticals	Randomized, open-label study (Phase 1 trial)	Jiangsu Province Hospital, China	From June through December 2019	A total of 24 healthy women were randomized. Eight women were assigned to each follitropin delta dose group (12, 18, and 24 µg). All 24 women completed the trial	21–40 years	Inclusion criteria: infertile women scheduled for IVF/ICSI cycles. Exclusion criteria: history/presence of any disease, including cardiovascular, musculoskeletal, immunological, endocrine, or metabolic disease; presence or history of severe allergy or anaphylactic reactions to any non-registered investigational drug were also ruled out; use of gonadotropin preparations within the 6 months prior to screening were excluded. Women were also not enrolled if they had participated in other clinical trials or donated blood in the past 4 weeks.	On the morning of the gonadotropin administration day, women were randomly assigned to receive a single dose of follitropin delta in a 1:1:1 ratio (12, 18, or 24 µg)	Not clearly reported. The study aims were to assess the pharmacokinetic characteristics, dose proportionality, and safety of follitropin delta in healthy Chinese women	The administration of single doses of follitropin delta to healthy Chinese women demonstrated dose-proportional pharmacokinetics over the dose range of 12–24 µg, and these doses were well tolerated.

AMH: Anti-Müllerian hormone, BMI: body mass index, CI: confidence interval, ICSI: intracytoplasmic sperm injection, IVF: in vitro fertilization, SD: standard deviation

### Qualitative analysis

#### Phase 1 RCTs

Shao et al. [27] conducted an open-label, randomized, parallel group design at Jiangsu Province Hospital, China, from June through December 2019. The participating women received two administrations of a 1-month depot formulation of triptorelin acetate depot (Decapeptyl®, Ipsen International) 3.75 mg to suppress endogenous release of FSH. The first dose of triptorelin was given 28 days before the administration of the first dose of follitropin delta (Rekovelle®, Ferring Pharmaceuticals) and the second dose was given 10 days prior to the first follitropin delta administration. On the morning of the gonadotropin administration day, 24 healthy Chinese women were randomized to receive a single dose of follitropin delta (12, 18, or 24 µg) administered subcutaneously in the abdominal region. A follow-up visit was scheduled 10 days after follitropin delta administration and an anti-FSH antibody assessment after 27 days. Following a single subcutaneous administration of follitropin delta 12, 18, or 24 µg, the maximum concentration observed (*C*_max_) (0.388, 0.677, and 0.825 ng/mL, respectively) and the area under the serum concentration–time curve from dosing to infinity (AUC_∞_) (41.3, 62.9, and 83.1 h·ng/mL, respectively) increased in a dose-proportional manner. The median time to reach *C*_max_ (*t*_max_) was 24 h, and the mean elimination phase half-life (*t*_½_) was in the range of 50.5–60.9 h. All treatment-related adverse events were categorized as mild, except for one case of urticaria from the follitropin delta 18-µg dose group which was considered moderate. Only one woman presented with elevation of alanine transaminase and aspartate aminotransferase at the follow-up visit, which was reported as a treatment-emergent adverse event. There were no injection-site reactions and none of the participants showed any confirmed presence of treatment-induced anti-FSH antibodies [27].

### Phase 2 RCTs

In 2014, Arce et al. [22] published the first randomized, controlled, assessor-blinded, AMH-stratified trial to evaluate the dose-response relationship of follitropin delta with respect to ovarian response in patients undergoing IVF/ICSI and to prospectively study the influence of initial AMH concentrations. On day 2–3 of the menstrual cycle, 265 eligible patients were randomly assigned, in a 1:1:1:1:1:1 ratio, to receive fixed daily subcutaneous injections of either 5.2 µg, 6.9 µg, 8.6 µg, 10.3 µg, or 12.1 µg of follitropin delta (FE 999,049; Ferring Pharmaceuticals), or 11 µg (150 IU) of follitropin alfa (Gonal-F®; Merck Serono). The reference arm (follitropin alfa, 11 µg) was included for external validity, and no statistical comparisons were contemplated. In patients treated with follitropin delta, the number of oocytes retrieved increased in a dose–dependent manner, from 5.2 ± 3.3 oocytes with 5.2 µg/d to 12.2 ± 5.9 oocytes with 12.1 µg/d. The slopes of the follitropin delta dose–response curves differed significantly between the two AMH strata, demonstrating that a 10% increase in dose resulted in 0.5 (95%CI 0.2, 0.7) and 1.0 (95%CI 0.7, 1.3) more oocytes in the low and high AMH stratum, respectively. Fertilization rate and blastocyst/oocyte ratio decreased significantly with increasing follitropin delta doses in both AMH strata. No linear relationship was observed between follitropin delta dose and number of blastocysts overall or by AMH strata. Five cases of OHSS were reported for the three highest follitropin delta doses and in the high AMH stratum [22].

Ishihara et al. [25] conducted a randomized, controlled, assessor-blind, AMH-stratified dose-response trial on 158 Japanese women aged between 20 and 39 years. On day 2–3 of the menstrual cycle, patients were randomized 1:1:1:1 to fixed daily subcutaneous injections of 6 µg, 9 µg, or 12 µg follitropin delta (Rekovelle®, 72 µg/2.16 mL; Ferring Pharmaceuticals), or 150 IU/d follitropin beta (Follistim®, 900 IU/1.08 mL; MSD) (reference arm for validation purposes). Randomization was stratified according to AMH levels at screening d (low 5.0–14.9 pmol/L; high 15.0–44.9 pmol/L). Among all women who started stimulation, the mean number (± SD) of oocytes retrieved in the 6 µg/d, 9 µg/d, and 12 µg/d follitropin delta groups were 7.0 ± 4.1, 9.1 ± 5.6, and 11.6 ± 5.6, respectively, and a significant dose-relation was established, which also remained significant within each AMH strata. The vital pregnancy rate per started cycle with follitropin delta was 19% for 6 µg/d, 20% for 9 µg/d, and 25% for 12 µg/d. The rate of early moderate/severe OHSS with follitropin delta was 8% for 6 µg/d, 8% for 9 µg/d, and 13% for 12 µg/d, with 82% of the cases in the high AMH stratum [25].

### Phase 3 RCTs

Four phase 3 RCTs have been published and included in the present systematic review. In all trials, a GnRH antagonist protocol was applied as strategy for inhibiting LH surge.

Nyboe Andersen et al. [15] conducted randomized, multicentre, assessor-blinded, non-inferiority trial (ESTHER-1) to compare the efficacy and safety of follitropin delta (Rekovelle®; Ferring Pharmaceuticals) with individualized dosing based on serum AMH and body weight, with conventional follitropin alfa (Gonal-F®; Merck Serono) dosing for ovarian stimulation in women undergoing IVF. A total of 1,329 women (aged 18–40 years) were randomized to receive follitropin delta (AMH < 15 pmol/L: 12 µg/d; AMH ≥ pmol/L: 0.10–0.19 µg/kg/d; maximum 12 µg/d), or follitropin alfa (150 IU/d for 5 days, potential subsequent dose adjustments; maximum 450 IU/d). The primary endpoints were the ongoing pregnancy rate and the ongoing implantation rate [15].

The ESTHER 2 trial was conducted by Bosch et al. [23] to study infertile patients who had undergone a first ovarian stimulation for IVF/ICSI cycle (cycle 1) in the ESTHER-1 [15] and who did not achieve ongoing pregnancy. Patients received during the further IVF/ICSI cycles the same gonadotropin treatments, i.e. follitropin delta (Rekovelle®; Ferring Pharmaceuticals) or alfa (Gonal-F®; Merck Serono). In cycle 2, 513 women were enrolled and exposed: 252 to follitropin delta and 261 to follitropin alfa. In cycle 3, 189 women were enrolled, of whom 188 were exposed: 95 to follitropin delta and 93 to follitropin alfa. The primary endpoint was the proportion of women with treatment-induced anti-FSH antibodies after up to two repeated cycles of ovarian stimulation. The incidence of treatment-induced anti-FSH antibodies with follitropin delta was 0.8% and 1.1% in cycles 2 and 3, respectively, which was similar to the incidence in cycle 1 (1.1%). No antibodies were of neutralizing capacity. Women with pre-existing anti-FSH antibodies were safely treated with follitropin delta without boosting an immune response [23].

Qiao et al. [24] conducted a randomized, controlled, multi-centre, assessor-blind trial conducted in 1009 Asian patients from mainland China, South Korea, Vietnam, and Taiwan, undergoing their first IVF/ICSI cycle. Randomization was stratified by age (< 35, 35–37, 38–40 years). The primary endpoint was ongoing pregnancy rate assessed 10–11 weeks after embryo transfer in the fresh cycle (non-inferiority limit − 10.0%; analysis adjusted for age stratum). The follitropin delta treatment consisted of a fixed daily dose individualised according to each patient’s initial AMH level and body weight (AMH < 15 pmol/l: 12 µg; AMH ≥ 15 pmol/l: 0.19 to 0.10 µg/kg; min-max 6–12 µg). The follitropin alfa dose was 150 IU/day for the first 5 days with subsequent potential dose adjustments according to individual response [24].

Ishihara and Arce [26] conducted a randomized, controlled, assessor-blind, multicentre, non-inferiority trial in 347 Japanese IVF/ICSI patients. They were randomized to individualized follitropin delta (AMH < 15 pmol/l: 12 µg/day; AMH ≥ 15 pmol/l: 0.10–0.19 µg/kg/day; minimum 6 µg/day; maximum 12 µg/day) or conventional follitropin beta (150 IU/day for the first 5 days, with potential subsequent dose adjustments). The primary endpoint was the number of oocytes retrieved with a pre-specified non-inferiority margin (− 3.0 oocytes). Non-inferiority was established for number of oocytes retrieved for individualized follitropin delta dosing compared with conventional follitropin beta dosing (9.3 versus 10.5 oocytes, respectively, lower boundary of 95%CI − 2.3) [26].

### Quantitative analysis

Data synthesis included three phase 3 RCTs [15, 24, 26]. Data from the ESTHER 2 trial [23] were not included in the meta-analysis because it was exclusively focused on the first cycle of randomization.

### Ovarian response endpoints

#### Duration of stimulation

All the three phase 3 RCTs included in the quantitative analysis reported the mean (± SD) duration of stimulation. Pooling of their results showed a significantly higher duration of stimulation in women treated with follitropin delta compared to those treated with follitropin alfa/beta [mean difference (MD) 0.34, 95%CI: 0.14, 0.53; *P* = 0.0008; I^2^ = 47%] (Fig. 1) (Table 2) [15, 24, 26].

**Table 2:** Summary of findings of phase 3 RCTs: follitropin delta compared to follitropin alfa/beta-

Outcomes	Anticipated absolute effects (95%CI)		Relative effect (95% CI)	Number of participants	Quality of the evidence (GRADE)	Comments
	Risk with follitropin alfa/beta	Risk with follitropin delta				
Duration of stimulation	The mean duration of stimulation in the control group ranged from 8.6 to 8.8 days	The duration of stimulation was longer in the group of women treated with follitropin delta compared to women treated with follitropin alfa/beta (MD 0.34, 95%CI: 0.14, 0.53; studies: 3)	//	2682	⊕⊕⊕⊕ High	//
Total dose of gonadotropin	The mean total dose of gonadotropin administered in the control group ranged from 103.7 to 149.9 µg	The total dose of gonadotropin administered was lower in the group of women treated with follitropin delta compared to women treated with follitropin alfa/beta (MD -37.07; 95%CI: -59.01, -15.13; studies: 3)	//	2682	⊕⊕⊕⊝ Moderate^1^	//
Number of oocytes retrieved	The mean number of oocytes retrieved in the control group ranged from 10.4 to 12.4 oocytes	The mean number of oocytes retrieved was comparable between study groups (MD -1.32; 95%CI:-2.64, -0.00; studies: 3)	//	2682	⊕⊕⊝⊝ Low^1,2^	//
Fertilization rate	The fertilization rate in the control group ranged from 56 to 64%	The fertilization rate did not differ between study groups (studies: 2)	//	//	⊕⊝⊝⊝ Very low^1,2,3^	Data of included studies could not be pooled because the denominator was not extractable
Number of day 3 embryos	The mean number of day 3 embryos obtained in the control group ranged from 5.7 to 8.7	The mean number of day 3 embryos obtained did not differ between study groups (studies: 2)	//	//	⊕⊝⊝⊝ Very low^1,2,3^	Data of included studies could not be pooled because the denominator was not extractable
Number of blastocysts	The mean number of blastocysts obtained in the control group ranged from 3.5 to 4.2	The mean number of blastocysts obtained did not differ between study groups (studies: 2)	//	//	⊕⊝⊝⊝ Very low^1,2,3^	Data of included studies could not be pooled because the denominator was not extractable
CPR	442 per 1348 (studies: 3)	455 per 1334 (studies: 3)	OR 1.06; 95%CI: 0.90, 1.24	2682	⊕⊕⊕⊝ Moderate^4^	//
OPR	373 per 1348 (studies: 3)	400 per 1334 (studies: 3)	OR 1.15; 95%CI: 0.90, 1.46	2682	⊕⊕⊕⊝ Moderate^4^	//
LBR	362 per 1348 (studies: 3)	394 per 1334 (studies: 3)	OR 1.18; 95%CI: 0.89, 1.55	2682	⊕⊕⊝⊝ Low^5^	//
LBR at 4 weeks after birth	327 per 1171 (studies: 2)	354 per 1164 (studies: 2)	OR 1.15; 95%CI: 0.81, 1.63	2335	⊕⊕⊝⊝ Low^5^	//
Adoption of preventive strategies for OHSS	87 per 1348 (studies: 3)	41 per 1334 (studies: 3)	OR 0.45; 95%CI: 0.30, 0.66	2682	⊕⊕⊝⊝ Low^5^	//
Early OHSS	86 per 1348 (studies: 3)	54 per 1334 (studies: 3)	OR 0.46; 95%CI: 0.31, 0.67	2682	⊕⊕⊝⊝ Low^5^	//
All forms of OHSS	100 per 1348 (studies: 3)	62 per 1334 (studies: 3)	0.61; 95%CI: 0.44, 0.84	2682	⊕⊕⊝⊝ Low^5^	//

OHSS: ovarian hyperstimulation syndrome, CPR: clinical pregnancy rate, OPR: ongoing pregnancy rate, LBR: live birth rate, MD: mean difference, OR: odds ratio, CI: confidence interval

1 downgraded one level due to the high statistical heterogeneity; 2 downgraded one level because the upper bound of the CI is equal to 0; 3 downgraded one level due to the impossibility of pooling data; 4 downgraded one level because only one study was adequately power for this outcome; 5 downgraded one level because none of the included studies was adequately powered for this outcome

GRADE Working Group grades of evidence

High certainty: we are very confident that the true effect lies close to that of the estimate of the effect

Moderate certainty: we are moderately confident in the effect estimate; the true effect is likely to be close to the estimate of the effect, but there is a possibility that it is substantially different

Low certainty: our confidence in the effect estimate is limited; the true effect may be substantially different from the estimate of the effect

Very low certainty: we have very little confidence in the effect estimate; the true effect is likely to be substantially different from the estimate of effect

#### Total dose of gonadotropin administered

**Fig. 2:**
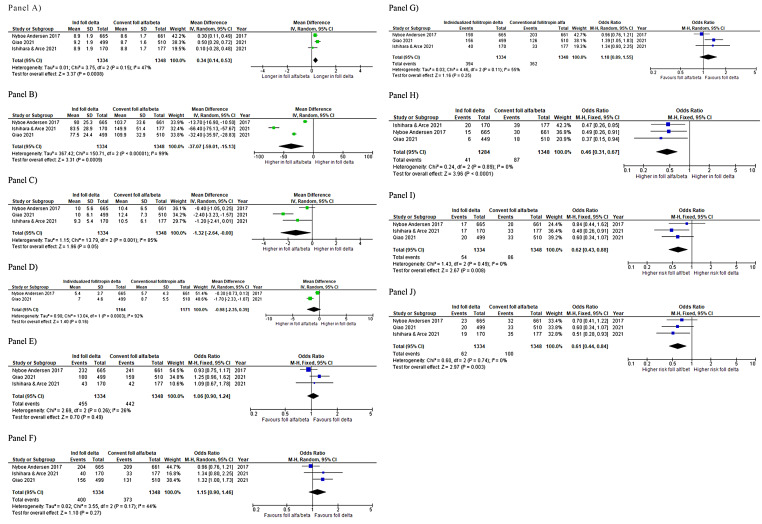
Meta-analysis of data extracted from phase 3 RCTs comparing individualized follitropin delta to conventional follitropin alfa/beta. Duration of stimulation (Panel A), total dose of gonadotropin administered (Panel B), number of oocytes retrieved (Panel C), number of day 3 embryos obtained (Panel D), clinical pregnancy rate (CPR) (Panel E), ongoing pregnancy rate (OPR) (Panel F), live birth rate (Panel G), rate of ovarian hyperstimulation syndrome (OHSS) preventive strategies adoption (Panel H), rate of early OHSS (Panel I), and rate of all forms of OHSS (Panel J)

The results of all the three phase 3 RCTs included in the quantitative analysis were pooled. The total dose of gonadotropin administered (µg) was significantly lower in women treated with follitropin delta compared to those treated with follitropin alfa/beta (MD -37.07, 95%CI: -59.01, -15.13; *P* = 0.0009; I^2^ = 99%) (Fig. 2) (Table 2) [15, 24, 26].

#### Number of oocytes retrieved

The results of three phase 3 RCTs were meta-analyzed. The number of retrieved oocytes per patient was not significantly different between groups (MD -1.32, 95%CI: -2.64, 0.00; *P* = 0.05; I^2^ = 85%) (Fig. 2) [15, 24, 26]. The analysis was also stratified according to the basal concentration of serum AMH. In women with serum AMH concentration < 15 pmol/l, the number of retrieved oocytes per patient was significantly higher in women treated with follitropin delta compared to women with follitropin alfa/beta (MD 1.05, 95%CI: 0.19, 1.92; *P* = 0.02; I^2^ = 56%) (Fig. 2) (Table 2) [15, 24, 26]. In women with serum AMH concentration ≥ 15 pmol/l, the number of retrieved oocytes per patient was significantly lower in women treated with follitropin delta compared to those treated with follitropin alfa/beta (MD -2.54, 95%CI: -3.95, -1.13; *P* = 0.0004; I^2^ = 77%) [15, 24, 26].

#### Fertilization rate

Two phase 3 RCTs reported the fertilization rate. Their results could not be pooled because the denominator could not be extracted. Both studies failed to show a significant difference in the fertilization rate between groups (Table 2) [15, 24].

#### Number of embryos obtained

Two RCTs reported the number of day 3 embryos obtained in the two study groups (Table 2) [15, 24]. The mean number of day 3 embryo obtained (independently from quality) was pooled. No difference between follitropin delta and follitropin alfa/beta was observed (MD -0.98, 95%CI: -2.35, 0.39; *P* = 0.0003; I^2^ = 92%) (Fig. 2) [15, 24]. Nyboe Anderson et al. [15] also did not observe any difference between treatment groups in the number of good quality day 3 embryos. Qiao et al. [24] conducted a sub-analysis according to the basal serum AMH value and observed: (i) in the group with basal serum AMH < 15 pmol/L, a significantly higher number of day 3 embryos obtained in women treated with follitropin delta compared to those treated with follitropin alfa (6.8 ± 3.7 and 5.6 ± 2.9 day 3 embryos, respectively (*P* = 0.0016)); (ii) in the group with basal serum AMH ≥ 15 pmol/L, a significantly higher number of day 3 embryos obtained in women treated with follitropin alfa compared to those treated with follitropin delta (9.6 ± 5.7 and 7.0 ± 4.8 day 3 embryos, respectively (*P* < 0.001) (Table 2) [24].

Two phase 3 RCTs reported the number of blastocysts obtained and did not observe differences between groups (Table 2) [15, 26]. These data could not be pooled because the denominator considered by Ishihara and Arce (i.e., the number of women with oocytes retrieved) was not extractable [26]. Ishihara and Arce [26] observed a number of blastocysts significantly lower in women treated with follitropin delta compared to women treated with follitropin beta (3.1 ± 2.7 and 4.2 ± 3.4 blastocysts, respectively, *P* < 0.001). The proportion of women who underwent blastocyst transfer was comparable between follitropin delta and follitropin beta treatment groups (79.4% vs. 79.7% for, respectively) [26]. Nyboe Anderson et al. [15] did not observe any difference between follitropin delta and follitropin beta treatment groups in the total number of blastocysts (3.3 ± 2.8 and 3.5 ± 3.2, respectively, *P* = 0.344) obtained as well as in the number of good quality blastocysts (2.0 ± 2.2 and 2.1 ± 2.4, respectively, *P* = 0.580) and in the number of cryopreserved blastocysts (1.9 ± 2.4 and 2.2 ± 2.6, respectively, *P* = 0.262).

### Reproductive outcomes

#### CPR

The data of three phase 3 RCTs were pooled. No difference between follitropin delta and follitropin alfa/beta was observed in CPR (OR 1.06; 95%CI: 0.90, 1.24; *P* = 0.49; I^2^ = 26%) (Fig. 2) (Table 2) [15, 24, 26].

#### OPR

Pooling of results of three phase 3 RCTs showed no difference in the OPR between women treated with follitropin delta compared to those treated with follitropin alfa/beta (OR 1.15; 95%CI: 0.90, 1.46; *P* = 0.27; I^2^ = 40%) (Fig. 2) (Table 2) [15, 24, 26].

#### LBR

The data of three phase 3 RCTs were pooled. The LBR was comparable between groups (OR 1.18; 95%CI: 0.89, 1.55; *P* = 0.25; I^2^ = 55%) (Fig. 2) (Table 2) [15, 24, 26].

Two RCTs reported the number of women with at least one live neonate 4 weeks after birth [15, 24]. Pooling of their results showed no difference between groups (OR 1.15; 95%CI: 0.81, 1.63; *P* = 0.43; I^2^ = 73%) (Table 2) [15, 24].

#### Cumulative endpoints

No studies reported cumulative success rates (i.e., CCPR, COPR, CLBR).

### Safety outcomes

#### OHSS

The rate of adoption of strategies to prevent OHSS development was significantly lower in women treated with follitropin delta compared to those treated with follitropin alfa/beta (3 studies, OR 0.45; 95%CI: 0.30, 0.66; *P* < 0.0001; I^2^ = 0%) (Fig. 2) (Table 2) [15, 24, 26]. The rate of both early OHSS and of all forms of OHSS was also significantly lower in the group of patients treated with follitropin delta compared to those treated with follitropin alfa/beta (OR 0.62; 95%CI: 0.43, 0.88; *P* = 0.008; I^2^ = 0% and OR 0.61; 95%CI: 0.44, 0.84; *P* = 0.003; I^2^ = 0%) (Fig. 2) (Table 2) [15, 24, 26].

### Risk of bias in included studies

#### Random sequence generation

**Fig. 3:**
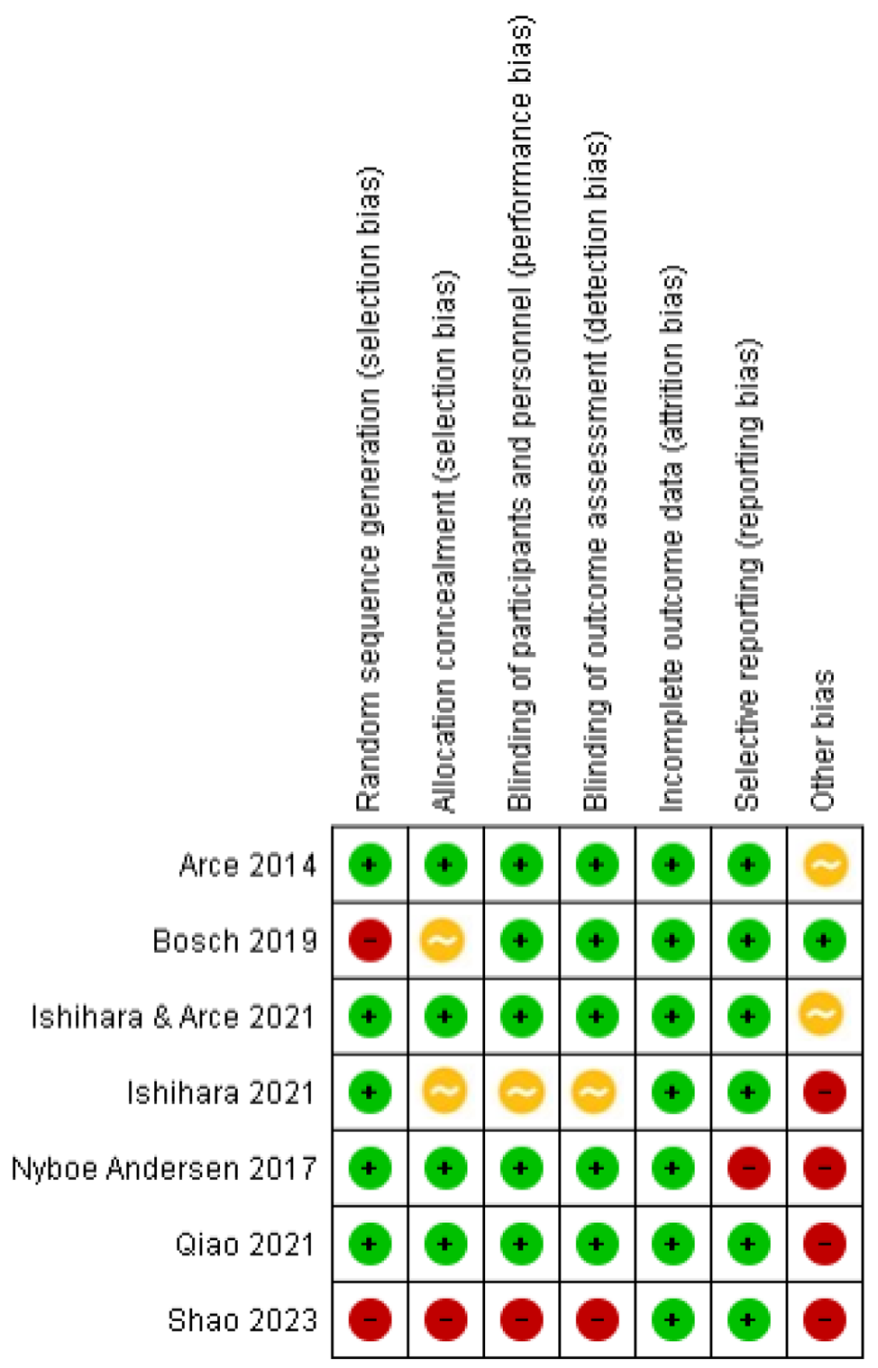
Risk of bias in included studies according to Higgins et al. [19]

The method of randomization was clearly stated in 5 clinical trials, and we assessed them a low risk of selection bias [15, 22, 24–26]. The method of randomization was less clear in the remaining trials (23, 27) (Fig. 3).

#### Allocation concealment

Four studies were rated as at low risk of selection bias related to allocation concealment as they used a central computer-generated randomization sequence [15, 22, 24, 26] (Fig. 3).

#### Blinding

Five trials were assessor-blind, and all investigators, embryologists and central laboratory personnel were blinded to treatment [15, 22, 23, 25, 26] (Fig. 3).

#### Incomplete outcome data

All the included trials reported no losses and had a cycle cancellation rate < 10% [15, 22–27] (Fig. 3).

#### Selective reporting

All the included trials were deemed at low risk of selective reporting [15, 22–27] (Fig. 3).

#### Other potential sources of bias

All the included trials were sponsored by Ferring Pharmaceuticals [15, 22–27] (Fig. 3).

### Quality of evidence

The quality of evidence provided by phase 3 RCTs is summarized in Table 2.

## Discussion

### Why it is important to do this review

The current study is the first systematic review of RCTs with meta-analysis aimed to provide type A evidence that follitropin delta, administrated using a AMH and BMI-based algorithm, is safer that other recombinant gonadotropin showing an efficacy profile not significantly different. Until now, all previous meta-analytic data have demonstrated a substantial similarity of the gonadotropins available in the market [28].

### Summary of the main results

Our results revealed that follitropin delta in fixed daily dose adjusted according to AMH level and body weight is not differently effective when compared to other recombinant gonadotropins, i.e., follitropin alfa and beta. In particular, no difference between two arms was observed in live birth, ongoing pregnancy and clinical pregnancy rate. Little but significant differences between two interventions were observed in the mean number of oocytes retrieved when data were analyzed according to AMH value with an amount of oocyte significantly higher and lower in the group with serum AMH concentration lower or higher than 15 pmol/L, respectively [15, 24, 26]. However, it should be recognized that the power of the included RCTs is insufficient to consider this sub-analysis reliable that should thus be considered “exploratory”. No differences in the number of day 3 embryos were observed by pooling the results of the two studies reporting this outcome [15, 24]. The data regarding the number of blastocysts obtained are contrasting. Nybo Andersen et al. [15] failed to observe any difference while Ishihara and Arce [26] observed a greater number of blastocysts in the group who received personalized follitropin beta treatment. Unfortunately, the different denominator considered by the two research groups makes it difficult to summarize these findings. On the other hand, notwithstanding the low grade of evidence, the safety profile of the two treatment arms was different. In particular, the rate of both early OHSS and of all forms of OHSS was significantly lower in the group of patients treated with follitropin delta compared to those treated with follitropin alfa/beta. Also, the adoption of OHSS preventive strategies was more frequent among women treated with follitropin alfa/beta.

Even if we planned at study design many sub-analyses/stratifications, we may confirm our main outcomes only for different recombinant gonadotropins (follitropin alfa vs. beta) and for different geographic areas (Asiatic vs. non-Asiatic populations) trough qualitative and quantitative analysis. Unfortunately, the very similar inclusion and exclusion criteria, and the availability of only sponsored studies by Ferring Pharmaceuticals make other sub-analyses not feasible.

### Agreements and disagreements with other studies

No previous systematic reviews of RCTs with meta-analysis are available in literature. At the moment, two systematic reviews are in progress on PROSPERO website and for both the anticipated completion date has been lasted. The first study protocol (CRD42022373275) was aimed to assess the efficacy and safety of follitropin delta in comparison with follitropin alfa in women undergoing ovarian stimulation for IVF/ICSI and intrauterine insemination cycles, whereas the second one (CRD42023399711) is an individual participant data meta-analysis of RCTs to assess the efficacy and safety of follitropin delta in comparison with other recombinant follitropins in infertile patients who received ovarian stimulation for IVF/ICSI cycles.

Our systematic review intercepted very few other non-randomized comparative trials. All were retrospective studies [29–31]. On the other hand, almost all prospective comparative studies available were post-hoc and/or sub-analysis of the RCTs included in the final analysis. For example, a recent sub-analysis of the GRAPE trial [23] demonstrated the non-inferiority of follitropin delta in comparison with follitropin alfa in terms of ongoing pregnancy (31.0% vs. 25.7% for follitropin delta vs. alfa, respectively; MD 5.1%, 95%CI: -1.3%, 11.5%) [32]. The clinical pregnancy rate and the live birth rate resulted also not significantly different [32]. Similarly to current data, individualized follitropin delta treatment resulted in fewer oocytes retrieved compared fixed follitropin alfa treatment, probably due to a mean reduction in patients with high ovarian reserve (AMH ≥ 15 pmol/L) of more than 3 oocyte per woman [32]. A lower incidence of early OHSS and/or preventive interventions was also observed (6.1% vs. 11.0%, for follitropin delta vs. alfa, respectively) [32].

### Quality of the evidence

Our data synthesis was performed on few original phase 3 parallel RCTs, even if they were large clinical trials including well-selected populations. However, almost all trials included in the current meta-analysis showed a high quality due to a low risk of bias, even if all were sponsored by the company that product the follitropin delta (Ferring Pharmaceutical).

### How the intervention works

The efficacy and the safety of the follitropin delta may be due to the specific pharmacokinetic and pharmacodynamic proprieties of the drugs due to its macro- and micro-heterogeneity in glycosylation, sialyation, and sulfation [33]. Follitropin delta contains a high sialic acid content (at both α2,3- and α2,6-linked sialic acids) that results in its increased charge and size compared with other follitropins, lower renal clearance and slower clearance from serum due to hepatic metabolism of α2,6-linked sialic acids [13, 14]. Specifically, the parent clone originally contained only α2,6-linked sialic acid but it was re-engineered by adding α2,3 linkages to reach the biopotency of follitropin alfa in rat model [33]. Thus, this specific glycoform composition of follitropin delta may exert clinical implications. However, it cannot be excluded that the results may be also due to the specific algorithm used for tailoring follitropin delta for ovarian stimulation in IVF/ICSI cycles that results to show a better performance than fixed standard doses [34]. This is more and more true considering that, various algorithms incorporating demographic/clinical and ovarian reserve data have been developed to optimize ovarian stimulation for IVF/ICSI cycles and to minimize the OHSS risk [9]. Several systematic reviews with meta-analyses have been conducted to test the algorithms to personalize FSH treatment for ovarian stimulation in IVF/ICSI cycles [35–37]. The most recent data synthesis of 4 RCTs for a total of 2823 patients, comparing an ovarian reserve test-based algorithm (basal FSH, AFC and AMH) with no algorithm, found a reduction of the risk of moderate/severe OHSS of about 40% (OR 0.58, 95% CI 0.34 to 1.00) with the use of the ovarian reserve test-based algorithm [37]. That results are in line with the current meta-analytic data.

### Overall completeness and applicability of evidence

The evidence appears to be not totally and broadly applicable for standard IVF and ICSI cycles. In particular, available data are exclusively limited to GnRH antagonist IVF/ICSI cycles. The analysis of the inclusion and exclusion criteria of the analyzed trials reveals that they were largely superimposable. The patient’ identikit of the subjects studied was an infertile patient at her first IVF/ICSI cycles, with a BMI for normal weight up to mild obesity, and with presumed normal ovarian response due to the inclusion of women with FSH levels lower than 15 UI and with normal menstrual cycles. Finally, all available evidence regards comparison between follitropin delta and other recombinant follitropins (alfa and beta follitropins), whereas wider analysis should be included also urinary gonadotropins as comparators [28].

Data on the use of personalized follitropin delta in long GnRH agonist protocol are very limited [21, 38, 39]. The first study was large phase 2 placebo-controlled double-blind RCT (RAINBOW trial) aimed to explore the efficacy and safety of choriogonadotropin beta as add-on treatment to follitropin delta in women undergoing ovarian stimulation in a long GnRH agonist protocol [21]. A sub-analysis of 104 patients allocated in the placebo arm showed data totally reassuring in terms of oocytes retrieved and blastocysts with an ongoing pregnancy and live-birth rate of 43% [38]. A total of 12 cases of OHSS were reported. Six cases were early mild/moderate OHSS (5.8%) and other 6 cases of late moderate/severe OHSS (5.8%) [38]. Data from a large prospective observational study also confirmed the good safety-efficacy profile of follitropin delta in GnRH agonist down-regulated IVF/ICSI cycles when used alone or in combination of other gonadotropins [39]. Finally, unpublished data from a large clinical trial (ClinicalTrial.gov NCT03809429, EudraCT number: 2017-002783-40) on 437 infertile patients without elevated markers of ovarian reserve showed no significant difference in efficacy and safety of follitropin delta for ovarian stimulation in IVF/ICSI cycles down-regulated with long GnRH agonist protocol when compared to short GnRH antagonist protocol (Table 3).

**Table 3:** Protocols for clinical trials intercepted about follitropin delta for ovarian stimulation in IVF/ICSI cycles. The search was performed on ClinicalTrials.gov, on clinicaltrialsregister.eu and on the national/international registries included on the WHO platform. Data are reported as detailed on the online register

Registry	Trial ID	Country (the first setting is reported)	Sample size (estimated for ongoing trial)	Study aim	Sponsor/funds	Status	Study start	Study competition (estimated)
ClinicalTrials.gov EU-CTR	NCT01426386 EudraCT number: 2011-000633-36	Belgium (multicenter)	265 Caucasian infertile eumenorrheic patients aged between 18 and 37 years, with BMI between 18.5 and 32.0 kg/m2, and without anovulatory PCOS stratified for AMH levels.	To investigate the effects of different doses of follitropin delta in women undergoing controlled stimulation for IVF/ICSI treatment on oocyte retrieved.	Ferring Pharmaceuticals	Completed, with results (published by Arce et al., 2014 [22])	2011-09-08	2013-03
ClinicalTrials.gov EU-CTR	NCT01956110 EudraCT number: 2013-001669-17	Belgium (multicenter)	1329 Caucasian infertile eumenorrheic patients aged between 18 and 40 years, basal FSH levels less than 15 UI/l, and BMI between 17.5 and 32.0 kg/m2.	To compare the efficacy and safety of follitropin delta with follitropin alfa having as primary endpoints the ongoing pregnancy and implantation rate in women undergoing ovarian stimulation for IVF/ICSI.	Ferring Pharmaceuticals	Completed, with results (ESTHER-1 TRIAL published by Nyboe Andersen et al., 2017 [15])	2013-10	2017-01-03
ClinicalTrials.gov EU-CTR	NCT01956123 EudraCT number: 2013-001616-30	Belgium (multicenter)	513 infertile patients included in a previous trial (NCT01956110) and failed to achieve an ongoing pregnancy but who undergone oocyte retrieval or had cycle cancellation prior to oocyte retrieval due to poor ovarian response or excessive ovarian response, in the previous cycle(s).	To investigate in repeated cycles the immunogenicity of follitropin delta having follitropin alfa as comparator arm.	Ferring Pharmaceuticals	Completed, with results (ESTHER-2 TRIAL published by Bosh et al., 2019 [23])	2014-03-26	2017-01-03
ClinicalTrials.gov	NCT02309671	Japan	159 infertile eumenorrheic patients aged between 20 and 39 years without PCOS.	To investigate the effects of different doses of follitropin delta having as comparator arm follitropin alfa in infertile women undergoing IVF/ICSI treatment.	Ferring Pharmaceuticals	Completed, with results (published by Ishihara et al. 2021 – phase 2 trial [25])	2014-12	2016-09
ClinicalTrials.gov	NCT03228680	Japan (multicenter)	373 Japanese infertile eumenorrheic patients aged between 20 and 40 years, basal FSH levels less than 15 UI/l, and BMI between17.5 and 32.0 kg/m2.	Non-inferiority study comparing follitropin delta and follitropin beta in terms of number of oocytes retrieved in women undergoing ovarian stimulation for IVF/ICSI.	Ferring Pharmaceuticals	Completed, with results (STORK trial published by Ishihara et al., 2021 [26])	2017-07-29	2019-07-08
ClinicalTrials.gov	NCT03296527	China (multicenter)	1011 Asian infertile eumenorrheic patients aged between 20 and 40 years, basal FSH levels less than 15 UI/l, and BMI between 17.5 and 32.0 kg/m2.	To demonstrate non-inferiority of follitropin delta in comparison with follitropin alfa in terms of ongoing pregnancy rate in women undergoing controlled ovarian stimulation for IVF treatment.	Ferring Pharmaceuticals	Completed, with results (GRAPE TRIAL published by Qiao et al., 2021 [24])	2017-12-01	2020-07-26
ClinicalTrials.gov ANZCTR	NCT03393780 Specific number not available	Australia (multicenter)	1018 infertile patients aged more than 18 years never treated with IVF/ICSI treatments	Observational study aimed to monitor the use in routine clinical practice of follitropin delta in patients who never underwent previous IVF/ICSI treatments.	Ferring Pharmaceuticals	Completed, with results (Blockeel et al., 2022 [39])	2018-03-16	2020-07-17
ClinicalTrials.gov EU-CTR	NCT03564509 EudraCT number: 2017-003810-13	Belgium (multicenter)	620 infertile patients aged between 30 and 42 years with AMH levels between 5.0 and 35.0 pmol/L, and without anovulatory PCOS.	To investigate the efficacy of FE 999,302 as add-on treatment to follitropin delta in women undergoing ovarian stimulation for IVF/ICSI in a long GnRH-a protocol, and to assess the safety profile, the potential immunogenicity, and the impact of body weight.	Ferring Pharmaceuticals	Completed, with results (Rainbow trial published by Fernández Sánchez et al., 2022 [21])	2018-05-14	2020-01-08
ClinicalTrials.gov	NCT03738618	United States (multicenter)	521 infertile eumenorrheic patients aged between 35 and 42 years, basal FSH levels less than 15 UI/l, and BMI between 17.5 and 38.0 kg/m2.	To investigate the safety and efficacy of follitropin delta in comparison to placebo arm.	Ferring Pharmaceuticals	Completed	2018-10-29	2020-12-21
ClinicalTrials.gov	NCT03740737	United States	579 infertile eumenorrheic patients aged between 18 and 34 years, basal FSH levels less than 15 UI/l, and BMI between17.5 and 38.0 kg/m2.	To investigate the safety and efficacy of follitropin delta in comparison to placebo arm.	Ferring Pharmaceuticals	Completed	2018-10-26	2020-11-20
ClinicalTrials.gov EU-CTR	NCT03809429 EudraCT number: 2017-002783-40	Austria (multicenter)	437 patients aged between 18 and 40 years with BMI between17.5 and 38.0 kg/m2 and without elevated markers of ovarian reserve.	To compare the efficacy and safety of follitropin delta for ovarian stimulation in IVF/ICSI cycles down-regulated with long GnRH agonist protocol vs. a short GnRH antagonist protocol.	Ferring Pharmaceuticals	Completed, with results^1^	2019-04-29	2022-02-16
ClinicalTrials.gov	NCT04150861	China (multicenter)	24 Chinese infertile patients eumenorrheic patients aged between 21 and 40 years, with BMI between 18.5 and 25.0 kg/m2, and without PCOS.	Open-label study to investigate the pharmacokinetic, safety and tolerability of follitropin delta in single subcutaneous dose in GnRH-agonist down-regulated IVF cycles.	Ferring Pharmaceuticals	Completed, with results (published by Shao et al., 2023 [27])	2019-06-23	2019-12-16
ClinicalTrials.gov	NCT04654039	Korea	600 Korean infertile patients who were prescribed follitropin delta for the first time.	Post-marketing surveillance study for re-examine the safety of follitropin delta to identify potential new adverse events, and to confirm its safety and effectiveness.	Ferring Pharmaceuticals	Recruiting	2020-10-26	2023-10-31
ClinicalTrials.gov	NCT04773353	India (multicenter)	220 Indian infertile eumenorrheic patients aged between 21 and 40 years, basal FSH levels less than 15 UI/l, and BMI between 17.5 and 32.0 kg/m2.	Non-inferiority study comparing follitropin delta and follitropin alfa in terms of ongoing pregnancy rate in women undergoing ovarian stimulation for IVF/ICSI.	Ferring Pharmaceuticals	Active, not recruiting	2021-12-03	2024-02
ClinicalTrials.gov EU-CTR	NCT05263388 EudraCT number: 2021-001785-38	Austria (multicenter)	300 infertile eumenorrheic patients aged between 18 and 40 years.	To compare the efficacy of follitropin delta (starting dose of 15 µg daily) in comparison with follitropin alfa (starting dose of 225 IU daily) for ovarian stimulation in IVF/ICSI cycles in terms of oocytes retrieved.	Ferring Pharmaceuticals	Recruiting	2022-07-10	2024-04-15
EU-CTR	EudraCT number: 2017-003810-13		773 subjects were screened of 619 subjects were exposed to investigational medicinal product (IMP): 515 to FE 999,302 and 104 to placebo.	To investigate the efficacy and safety of FE 999,302 as add-on treatment to follitropin delta in women undergoing ovarian stimulation in a long GnRH-a protocol	Ferring Pharmaceuticals	Completed with results unpublished.	2019-10-21	2020-01-30
ReBec	U1111-1247-3260	Brasil	44 (intervention group) vs. 280 (historical control group) infertile eumenorrheic patients aged between 18 and 40 years, basal FSH levels less than 15 UI/l, and BMI between 17.5 and 32.0 kg/m2.	Prospective interventional study with historical control aimed to evaluate the effect of hp-FSH addition at standard staring doses (150 UI daily) in patients who receive follitropin delta.	Ferring Pharmaceuticals	Not yet recruiting	2020-01-03	2020-12-31

^1^open-label study. ^2^ results are available at https://www.clinicaltrialsregister.eu/ctr-search/trial/2017-002783-40/results

All data included regard GnRH-ant down-regulated IVF/ICSI cycles, if it is not specifically reported a different protocol. The dates correspond to those primarily reported in ClinicalTrial.gov website. No trial recorded was found using “FE 999049”, “follitropin delta”, “gonadotropin delta” and “rekovelle” as key words on the following registries: ChiCTR (Chinese Clinical Trial Registry), CRIS (Clinical Research Information Service for clinical trials conducted in Korea), CTRI (Clinical Trials Registry - India), RPCEC (Cuban Public Registry of Clinical Trials), IRCT (Iranian Registry of Clinical Trials), ISRCTN (International Standard Randomised Controlled Trial Number), ITMCTR (International Traditional Medicine Clinical Trial Registry), JRCT (Japan Registry of Clinical Trials), LBCTR (Lebanese Clinical Trials Registry), TCTR (Thai Clinical Trials Registry), PACTR (Pan African Clinical Trial Registry), REPEC (Peruvian Clinical Trial Registry), and SLCTR (Sri Lanka Clinical Trials Registry)

AMH: anti-Mullerian hormone, ANZCTR: Australian New Zealand clinical trial register, BMI: body mass index, DRKS: German Clinical Trials Register, EU-CTR: European Union Clinical Trials Register, FSH: follicle stimulating hormone, GnRH-a: gonadotropin releasing hormone agonist, GnRH-ant: gonadotropin releasing hormone antagonist, hp: high purified, ICSI: intracytoplasmic sperm injection, IVF: in vitro fertilization, PCOS: polycystic ovary syndrome, ReBec: Brazilian Clinical Trials Registry

Data on potentially high-responders are also limited, even if they seem to suggest that the follitropin delta, given according to specific BMI and AMH-based algorithm, is safer than follitropin alfa [15, 40–43]. In fact, in patients with polycystic ovarian morphology, the incidence of early moderate/severe OHSS and/or preventive interventions for early OHSS was 7.7% with individualized follitropin delta and 26.7% with conventional follitropin alfa [15]. The safety of the protocol based on individualized follitropin delta has been also explored in 64 infertile women with PCOS, diagnosed according to Rotterdam criteria [42]. A further sub-analysis of the ESTHER-1 [15], including 153 potential high responders identified on the basis of baseline serum AMH levels (above 35 pmol/l), showed that patients treated with individualized follitropin delta had fewer dominant follicles and oocytes retrieved than patients who received conventional follitropin alfa [41]. The OHSS risk, the incidence of early moderate or severe OHSS, and the use of preventive interventions for early OHSS resulted three times lower in the individualized follitropin delta arm [41]. These data confirm a previous sub-analysis of the ESTHER 1 and ESTHER 2 showing that the greatest benefit in terms of OHSS risk and prevention was observed in patients in the highest AMH quartile (≥ 25.35 pmol/l) who received individualized follitropin delta [40]. Of note, the ongoing pregnancy rate per started cycle after fresh blastocyst transfer was 28.2% and 24.0% for follitropin delta and alfa, respectively [41]. This result was probably due to the lower incidence of patients in follitropin delta group with serum progesterone levels higher than 3.18 nmol/l [41].

### Strengths and limitations

Our study has several strengths. First, our protocol study followed closely the PRISMA 2020 statement [16] and the PICO model [17]. Second, an extensive search for RCTs on electronic databases was performed to intercept not only published studies but also on almost all international and national registries for clinical studies protocols (Table 3). Third, large RCTs studying well-studied populations of infertile women were included in the final analysis. Moreover, current study has also many limitations. The number of the clinical trials included in the quantitative analysis was very little [15, 24, 26]. All included trials were industry sponsored and this may have introduced a bias in favor of the gonadotropin produced by the sponsor [43]. Thus, no subgroup analysis was feasible for the lack of independent non-sponsored studies. To this regard, a previous meta-analysis [28] showed that that live birth and clinical pregnancy rate were lower for r-FSH (follitropin alfa and beta) compared to urinary FSH in the Ferring Pharmaceuticals sponsored trials after subgroup analyses for pharmaceutical sponsoring. The design of the phase 3 RCTs also deserves to be commented. In particular, according to detractors, the fair comparison between follitropin delta and follitropin alfa requires individualized starting dose in both arms, using available nomograms for FSH, whereas in all published RCTs individualized starting dose was not allowed for follitropin alfa [34]. Finally, another limitation regards the safety of follitropin delta. The risk reduction of OHSS resulted to have a low grade of evidence. In addition, following original data, preventive interventions to reduce OHSS risk were also included as “marker” for OHSS, and this may confound the overall data. In fact, the study protocols and original papers did not specify when the preventive interventions were performed suggesting that they were deliberatively employed. Finally, for many important outcomes *(i.e.*, the total dose of gonadotropins, the number of retrieved oocytes) we observed a high statistical heterogeneity. As regards the total dose of gonadotropins, this heterogeneity is due to the approach adopted by Ishihara and Arce [26] which resulted in a much more pronounced dose difference between the two study arms compared to the other two trials [15, 24]. As stated by the authors themselves, in fact, the daily dose was adjusted in 46.3% of the women treated with follitropin beta, with the majority of adjustments being a dose increase on stimulation day 6 [26]. Regarding the number of oocytes retrieved, the high heterogeneity is due to the results of the study by Qiao et al. [24] which showed a much more pronounced difference in the number of oocytes than the other included studies. Looking further into these results [24], it can be noted that, while in the group of women with reduced ovarian reserve a significantly higher number of oocytes was retrieved in the arm treated with follitropin delta, in the group of women with good ovarian reserve a significantly higher number of oocytes was retrieved in the group of women treated with follitropin alfa. The result is due to imbalance in the distribution of the population since only approximately 1 in 5 patients had baseline AMH values < 15 pmol/l [24]. However, a random-effects meta-analysis was used to incorporate heterogeneity among studies.

### Implications for practice

Our findings suggest that follitropin delta, administrated in a personalized fashion and compared to other recombinant gonadotropins, is similarly effective but safer, for ovarian stimulation in IVF/ICSI cycles in terms of reduction OHSS risk. However, from a clinical point of view, these results may be transferred exclusively to populations with the same characteristics as those selected in the included trials and, in particular, to potentially normo-responder patients who receive their first ovarian stimulation GnRH antagonist IVF/ICSI cycle. In addition, efficacy data cannot be considered definitive because cumulative reproductive findings are totally lacking. To this regard, the CLBR per oocyte retrieval is considered more meaningful than LBR “per cycle” or “per ET episode” as it is a much better indicator of quality and success in IVF in its totality as the use of extended embryo culture as well as cryopreservation have become an integral part of IVF [44, 45]. Unfortunately, the three phase 3 RCTs included in the present meta-analysis did not report this outcome. Investigating possible differences in CLBR between the two study groups should thus be considered a priority in future studies.

Based on the available data, however, some speculations can be made. A growing body of evidence demonstrates a positive correlation between the number of oocytes retrieved and CLBR [5]. On the other hand, a high number of oocytes also increases the likelihood of developing OHSS [46]. Herein, we observed that, in women with serum AMH ≥ 15 pmol/l, the follitropin alfa/beta treatment was associated with both a higher number of retrieved oocytes and a higher incidence of OHSS compared to the follitropin delta treatment. It could therefore be hypothesized that, in the group of women with good/high ovarian reserve, the use of follitropin alfa/beta might be associated with an increase in CLBR. Should this theory be confirmed by future studies, the availability of these different drugs would offer a further opportunity to personalize IVF treatment. More specifically, having more oocytes might represent an additional value for increasing CLBR, mainly in those IVF Units where the “freeze all approach” for preventing OHSS is a well-established strategy. On the other hand, reducing the risk of OHSS might be crucial for those IVF Units that prefer to limit freezing procedures in favour of fresh embryo transfers.

### Implications for research

The main shortage of the current evidence is the absence of data regarding both the number and quality of frozen embryos and the success rates. Filling these gaps should therefore be considered a priority for future research. A further important issue is to clarify if the better safety-efficacy profile of the follitropin delta is due to the specific pharmacokinetic/dynamic proprieties of the drugs or to the efficacy of the algorithm used for personalizing its administration [34]. Unfortunately, that algorithm was explored and validated only for follitropin delta. It’s obvious that available clinical trials are not head-to-head comparisons between different gonadotropins. New data should compare different gonadotropins using the same strategy of administration (personalized or fixed) in order to obtain “pure” comparative results. However, it should be needed to explore the safety and the efficacy of the same algorithm, based on pre-treatment AMH levels and body weight, also for the other gonadotropins. Similarly, it should be interesting to evaluate the administration of follitropin delta using also fixed protocol or protocols based on clinician’ decision. On the other hand, it is possible also to hypothesize that further algorithms may more and more optimize the safety-profile of the follitropin delta or that the specific pharmacokinetic/dynamic proprieties of each gonadotropin may influence the efficacy of each specific algorithm.

In consideration of the very great number of IVF/ICSI cycles worldwide scheduled using a long GnRH agonist protocol, it should be defined on the safety and efficacy of follitropin delta also in GnRH agonist down-regulated IVF/ICSI cycles. More research is also needed to test the efficacy of the algorithm for lean and moderately or severely obese patients, and to confirm the efficacy of personalized follitropin delta in patients at risk for high or poor response, such as in patients who receive previous IVF/ICSI cycles of gonadotropins, not exclusively after one ovarian stimulation with follitropin delta [23]. The selection and the inclusion of eumenorrheic women presumably excluded a large proportion of patients with polycystic ovary syndrome (PCOS) and the safety-efficacy of personalized follitropin delta should be specifically studied in further trials. The evaluation of the tailoring of follitropin delta administration in subsequent IVF cycles, especially in case of poor-response or of hyper-response in the previous cycle(s) is certainly a future study aim. The preference of patients and of clinicians for a particular type of gonadotropin is also an important issue, to date too little evaluated in the literature any trial. To date, we did not know the preference profile for patients according to follitropin delta or other gonadotropins, and the very low number of non-sponsored studies may suggest a low preference of the clinicians in the use of gonadotropin based on specific algorithm. Finally, very few and sparse data are available about the long-term safety for the mother and the baby [47], and an international register might be useful to monitor these issues.

## Conclusion

The current systematic review of RCTs with meta-analysis showed that the treatment with follitropin delta, administrated using an AMH- and body weight-based algorithm, was associated with a lower rate of all forms of OHSS compared to other recombinant gonadotropins, in potentially normo-responder patients who receive ovarian stimulation in GnRH antagonist IVF cycles.We observed a comparable rate of reproductive success per cycle or per single episode of ET between the group treated with follitropin delta and that treated with follitropin alfa/beta. However, the absence of cumulative data does not allow definitive conclusions to be drawn regarding the comparison of the effectiveness of the two treatments. Further studies that, on the one hand, confirm the difference in the safety profile and its clinical entity between the two treatments and, on the other, that fill the absence of data relating to cumulative success rates are warranted.

## Data Availability

The data underlying this article will be shared on reasonable request to the corresponding author.

## Abbreviations

AMH: Anti-Müllerian hormone
ANZCTR: Australian New Zealand clinical trial register
BMI: Body mass index
ChiCTR: Chinese Clinical Trial Registry
CI: Confidence interval
CPR: Clinical pregnancy rate
CRIS: Clinical Research Information Service for clinical trials conducted in Korea
CTRI: Clinical Trials Registry – India
DRKS: German Clinical Trials Register
EU-CTR: European Union Clinical Trials Register
FSH: Follicle stimulating hormone
GnRH: Gonadotropin releasing hormone
GRADE: Grading of Recommendations Assessment, Development and Evaluation
Hp: High purified
ICSI: Intracytoplasmic sperm injection
IRCT: Iranian Registry of Clinical Trials
ISRCTN: International Standard Randomized Controlled Trial Number
ITMCTR: International Traditional Medicine Clinical Trial Registry
ITT: Intention-to-treat
IVF: In vitro fertilization
JRCT: Japan Registry of Clinical Trials
LBCTR: Lebanese Clinical Trials Registry
LBR: Live birth rate
LH: Luteinizing hormone
MD: Mean difference
OHSS: Ovarian hyperstimulation syndrome
OPR: Ongoing pregnancy rate
OR: Odds ratio
PACTR: Pan African Clinical Trial Registry
PICO: Population, Intervention, Comparison, Outcome
PCOS: Polycystic ovary syndrome
PRISMA: Preferred Reporting Items for Systematic Reviews and Meta-Analyses
ReBec: Brazilian Clinical Trials Registry
REPEC: Peruvian Clinical Trial Registry
RPCEC: Cuban Public Registry of Clinical Trials
SLCTR: Sri Lanka Clinical Trials Registry
TCTR: Thai Clinical Trials Registry
WHO: World Health Organization
